# Developmental age and clinical illness drive *in vivo* variability of CYP1A2 ontogeny and caffeine metabolism in preterm neonates

**DOI:** 10.3389/fphar.2026.1823969

**Published:** 2026-05-20

**Authors:** Michelle Chenghuazou Wang, Victoria A. Sealey, Mehzabin Rahman, Malaika Ngugama, Amarine Trolat, Leanne Wybenga-Groot, Michael F. Moran, Jean Dinh, Tamorah Rae Lewis

**Affiliations:** 1 Division of Clinical Pharmacology and Toxicology, Department of Paediatrics, The Hospital for Sick Children, Toronto, ON, Canada; 2 Department of Pharmacology and Toxicology, University of Toronto, Toronto, ON, Canada; 3 Program in Translational Medicine, SickKids Research Institute, Toronto, ON, Canada; 4 SPARC BioCentre, The Hospital for Sick Children, Toronto, ON, Canada; 5 Program in Molecular Medicine, The Hospital for Sick Children, Toronto, ON, Canada; 6 Department of Molecular Genetics, University of Toronto, Toronto, ON, Canada; 7 Certara UK Limited, Sheffield, United Kingdom; 8 Division of Neonatology, Department of Paediatrics, The Hospital for Sick Children, Toronto, ON, Canada

**Keywords:** CYP1A2, cytochrome P450, drug metabolism, neonate - age, ontogeny, pharmacokinetics, physiologically based pharmacokinetic (PBPK) modeling, preterm infant

## Abstract

**Background:**

Caffeine is the standard therapy for apnea of prematurity and used near-universally in preterm infants. Interindividual variability in clearance, exposure, and clinical responses persists in neonates. CYP1A2 is the primary enzyme responsible for caffeine metabolism in adults, but the enzyme activity in neonates has historically been considered negligible.

**Methods:**

Scavenged plasma samples from neonates receiving caffeine therapy in a neonatal intensive care unit were analyzed to quantify caffeine and paraxanthine concentrations. The caffeine metabolic ratio (CMR) was used as a functional biomarker of *in vivo* CYP1A2 activity. Associations between CMR, postmenstrual age, and clinical covariates were evaluated using univariate analyses, linear mixed-effects modeling, and longitudinal analyses. To compare our data to model predictions, caffeine concentrations were simulated in preterm infants using two previously published physiologically based pharmacokinetic (PBPK) models: the default Simcyp preterm infant model and a 2025 modified model.

**Results:**

Thirty-one neonates (186 plasma samples) were recruited for this study. Paraxanthine was detectable in all samples, demonstrating measurable CYP1A2 activity. CMR increased with postmenstrual age even after adjusting for clinical covariates (*β* = 0.05, *p* = 0.01). Interindividual variability was observed and longitudinal analyses showed heterogeneous CMR trajectories, indicating modulation by clinical factors beyond age alone. Both PBPK models tested demonstrated systematic overprediction of caffeine exposure, consistent with underestimation of clearance, although the modified model showed better concordance with observed data.

**Conclusion:**

These findings provide the first *in vivo* evidence of quantifiable CYP1A2 activity in neonates and demonstrate the feasibility of using caffeine as a probe drug to study enzyme ontogeny. Current published PBPK models do not accurately capture caffeine concentrations in our cohort, likely reflecting differences between our population and the preterm models. Integrating empirically derived neonatal pharmacokinetic data into PBPK models that more accurately reflect a preterm NICU population may better inform individualized dosing based on developmental age and clinical illness severity. By anchoring neonatal drug dosing in empirically derived physiology rather than adult extrapolation, these models have the potential to transform dosing practice and advance pharmacoequity for one of the most vulnerable and historically understudied populations.

## 1 Introduction

Prematurity remains a leading cause of neonatal morbidity and mortality worldwide, with approximately one in ten infants born preterm (Ohuma et al., 2023). Apnea of prematurity (AOP) is one of the most common complications affecting preterm neonates, particularly those born earlier than 28 weeks gestational age (GA) (Eichenwald and Committee on Fetus and Newborn, American Academy of Pediatrics, 2016; Mathew, 2011). AOP is characterized by recurrent pauses in breathing due to immature brain development and is often accompanied by intermittent hypoxemia and bradycardia. Caffeine citrate (hereinafter referred to as caffeine) is the standard of care for AOP and is among the most frequently prescribed medications in neonatal intensive care units (NICUs) (Aranda et al., 2010; Stark et al., 2022). Widespread use of caffeine is supported by robust clinical evidence demonstrating reductions in apneic events, decreased need for mechanical ventilation, and improvements in long-term neurodevelopmental outcomes (Aranda et al., 2010; 1977; Ostrem et al., 2025; Schmidt et al., 2012; 2007; 2006).

Despite caffeine’s widespread use and overall favorable safety profile (Schmidt et al., 2012; 2007; 2006), substantial interindividual variability in caffeine exposure and response persists among neonates (Charles et al., 2008; Lee et al., 1997; Long et al., 2021). Current dosing strategies rely on body weight (Anderson and Holford, 2009; Eichenwald and Committee on Fetus and Newborn, American Academy of Pediatrics, 2016; Long et al., 2021), which does not adequately reflect the rapid and heterogeneous developmental changes in physiology that occur during the neonatal period among a range of GA at birth (Allegaert and van den Anker, 2014; Anderson and Holford, 2009; Aranda and Beharry, 2020; Gao et al., 2021; Kearns et al., 2003; van Groen et al., 2021). As a result, some infants may experience subtherapeutic caffeine exposure, manifesting as persistent apnea (Charles et al., 2008; He et al., 2021), while others may experience adverse effects such as tachycardia or feeding intolerance (Aranda et al., 2010; Aranda and Beharry, 2020). Understanding the factors underlying interindividual variability in caffeine exposure is critical for optimizing caffeine therapy in preterm neonates.

Developmental changes in drug disposition during early life are driven by rapid maturation of organ systems and drug-metabolizing enzymes, with considerable interindividual variability influenced by biological maturity and age (Abduljalil et al., 2020; Anderson and Holford, 2009; Charles et al., 2008; Dai et al., 2024; Ide et al., 2024; Thomas et al., 2025), illness severity (Allegaert and van den Anker, 2014; Aranda and Beharry, 2020; Gao et al., 2021), nutritional status (Blake et al., 2006), exposure to concomitant medications (van Groen et al., 2021), and genetic variability in drug-metabolizing enzymes (He et al., 2021; Kearns et al., 2003; Long et al., 2021; Thorn et al., 2012). In particular, the ontogeny of drug-metabolizing enzymes plays a central role in shaping neonatal pharmacokinetics (Hines and McCarver, 2002; Kearns et al., 2003). Hepatic enzyme systems undergo rapid and non-linear maturation after birth, and the relative contribution of individual metabolic pathways differs substantially from that observed in older children and adults (Hines, 2007; Sonnier and Cresteil, 1998; van Groen et al., 2021). Consequently, extrapolation of adult pharmacokinetic knowledge to neonates is often inaccurate without direct developmental data from the youngest patient populations (Anderson and Holford, 2009; van den Anker et al., 2018).

Caffeine is primarily metabolized by hepatic cytochrome P450 1A2 (CYP1A2) to paraxanthine and is the major determinant of caffeine clearance (Darnaud et al., 2023; Fuhr et al., 2007; 1996; Perera et al., 2012; Tian et al., 2019). In adults, interindividual variability in caffeine pharmacokinetics is driven by pharmacogenetic variation and environmental factors such as smoking, diet, and drug–drug interactions (Darnaud et al., 2023; Fuhr et al., 2007; Perera et al., 2012; Sachse et al., 1999; Thorn et al., 2012; Tian et al., 2019). In contrast, neonatal caffeine metabolism remains poorly understood and has historically been assumed to involve negligible CYP1A2 activity. This view stems from limited detection of caffeine metabolites in neonatal plasma and minimal *in vitro* hepatic CYP1A2 expression until late infancy (Aldridge et al., 1979; He et al., 2021; Lee et al., 1997; Sonnier and Cresteil, 1998; Subash and Prasad, 2024). As a result, neonatal caffeine clearance has traditionally been characterized as predominantly renal, with minimal hepatic contribution, an assumption reflected in contemporary pharmacokinetic models emphasizing renal function early in development (Aranda and Beharry, 2020; Lee et al., 1997; Thomas et al., 2025). However, since CYP protein expression does not necessarily correlate with functional activity, it is important that CYP1A2 ontogeny in neonates is directly characterized *in vivo*, filling a critical knowledge gap (Pearce et al., 2016).

In adults, the caffeine metabolic ratio (CMR), defined as the paraxanthine-to-caffeine ratio, is a well-validated biomarker of *in vivo* CYP1A2 activity (Darnaud et al., 2023; Fuhr et al., 1996; Perera et al., 2012; Tian et al., 2019). The routine clinical use of caffeine in neonates provides a unique opportunity to leverage CMR as an *in vivo* biomarker of CYP1A2 activity during early life (Aranda and Beharry, 2020; Eichenwald and Committee on Fetus and Newborn, American Academy of Pediatrics, 2016; Stark et al., 2022). By using CMR, this is a novel strategy to define CYP1A2 ontogeny in neonates, identify clinical covariates of caffeine metabolism (Kearns et al., 2003; van Groen et al., 2021), improve mechanistic models of neonatal drug disposition (Dinh et al., 2023; Thomas et al., 2025), and build on precedent for using clinically indicated drugs as developmental enzyme probes, as demonstrated previously for CYP2C19 using pantoprazole (Ward et al., 2010; Ward and Kearns, 2013).

Physiologically based pharmacokinetic (PBPK) models provide a mechanistic framework to integrate the development of a preterm population, however, their predictive performance in neonates is fundamentally limited by sparse *in vivo* data describing enzyme ontogeny (Abduljalil et al., 2020; Dinh et al., 2023; van den Anker et al., 2018). In particular, ontogeny functions of drug-metabolizing enzymes incorporated into pediatric PBPK models have largely relied on *in vitro* data (mRNA, protein levels, and cell experiments), which incompletely capture functional enzyme activity during early development (Salem et al., 2014). Drug concentrations of probe CYP substrates that can be obtained during routine neonatal care from infants represents a critical, underutilized source of information that has the potential to improve PBPK models (Allegaert and van den Anker, 2014; Dinh et al., 2023; Kearns et al., 2003). The clinical data obtained from scavenged samples can be used to validate existing models and describe plausible ontogeny functions.

This study aims to characterize *in vivo* CYP1A2 ontogeny in neonates who were receiving standard dosing of caffeine, using CMR as a functional biomarker of enzyme activity. By defining the developmental trajectory of CYP1A2, this work provides insight into the maturational and clinical factors that contribute to interindividual variability in neonatal caffeine exposure and response. Elucidation of these key factors will also help identify key covariates for incorporation into PBPK models tailored to preterm neonates. By directly comparing clinical pharmacokinetic data with model-based simulations, this study also seeks to assess the validity of current neonatal PBPK frameworks and identify areas where mechanistic refinement may be required.

## 2 Materials and methods

### 2.1 Study design

This single-center, prospective observational cohort study was conducted in neonates recruited from the tertiary/quaternary NICU at The Hospital for Sick Children (SickKids) in Toronto, Canada. The study protocol was submitted to the SickKids Research Ethics Board (REB) and approved on 3 January 2024 (1000081067). The study was designed and conducted according to the Declaration of Helsinki and relevant instructional policies. Written informed consent was obtained from the parents or guardians of all patients before study procedures.

### 2.2 Participants

Patients were recruited for this study between January and June 2025. Patients were eligible for this study if they were (1) prescribed caffeine during their NICU admission at SickKids as per standard of care, (2) received at least 7 doses of intravenous or oral caffeine prior to the study (refer to Section 2.3 for details), and (3) received at least one clinical blood draw during their treatment window. Patients were excluded from this study if (1) they received a jejunostomy or duodenostomy and received caffeine orally because they would have presumed abnormal absorption (Aranda and Beharry, 2020; Olivares-Morales et al., 2015; Perera et al., 2012), (2) the clinical care team did not feel it was appropriate to approach the parents or guardians of the patient, or (3) the parents or guardians were unable to provide informed consent.

### 2.3 Clinical specimens

All participants received a loading dose of 10 mg/kg of caffeine intravenously on their date of birth, followed by a daily maintenance dose of 5 mg/kg (3–10 mg/kg depending on clinical status), as per standard of care. Maintenance doses were given intravenously as boluses or orally based on the clinical care team’s judgements. To minimize harm to participants, blood samples were scavenged from leftover clinical blood draws. Plasma samples were scavenged for caffeine and metabolite quantification while participants were receiving caffeine. At least one plasma sample was scavenged for each participant. For participants with prolonged hospital admissions, longitudinal plasma samples were obtained on a weekly basis, depending on frequency of labs and plasma volume sufficiency.

### 2.4 Caffeine and paraxanthine plasma sample preparation

Caffeine, paraxanthine, caffeine-d9, and paraxanthine-d3 were purchased from LGC (TRC-C080100-1G, TRC-C080102-2.5MG, TRC-P192500-25MG & TRC-P192503-1MG; LGC, ON, CA). HPLC-grade solvents and formic acid were purchased from Fisher. Since endogenous caffeine is often detectable in neonatal plasma (e.g., via breast milk transfer), a surrogate matrix of porcine plasma (IGPCPLANAE50ML, Innovative Research Inc., Novi MI, USA) was used for calibration curve preparation as it provides a true blank matrix and exhibits broadly comparable protein content, lipid composition, and ionization behaviour, making it a suitable surrogate for human plasma in LC–MS assays (McLaughlin, 2022; McLaughlin et al., 2025). Matrix equivalence between porcine and human plasma was also assessed during method validation. Standards and samples were extracted following the same procedure. Briefly, 10 µL of plasma was added to a microcentrifuge tube containing 1 ng of caffeine-d9 and 0.5 ng of paraxanthine-d3 internal standard in methanol. 1.2 mL of acetonitrile (ACN) was added to each tube followed by vortexing (30 s) and centrifugation (20,000 g, 15 min, 4 °C). Supernatants were transferred to conical glass tubes containing 3 mL ACN and evaporated to dryness under a gentle flow of nitrogen at 35 °C. Dried residues were reconstituted in 120 µL of methanol, centrifuged (2200 RPM, 2 min, RT) and supernatants transferred to a glass insert for analysis by LC-MS/MS.

### 2.5 Quantification by LC-MS/MS

Caffeine and paraxanthine were quantified by liquid chromatography-tandem mass spectrometry (LC-MS/MS) using a QTRAP 5500 triple quadrupole mass spectrometer (SCIEX, Concord, ON, CA) in positive electrospray ionization (ESI) MRM data acquisition mode, with an Agilent 1200 HPLC (Agilent Technologies, CA, USA). The targeted mass (m/z) were 195.1⟶83.0 for caffeine and 181.1⟶68.9 for paraxanthine. Chromatography was performed on a Kinetex C18 column (50 mm × 3 mm, 2.6 µm, #00B-4496-Y0; Phenomenex, CA, USA). HPLC flow was maintained at 500 μL/min with a gradient consisting of: A = Water+ 0.1% formic acid and B = ACN + 0.1% formic acid with a total run time of 8.5 min. Initial conditions were 95% A and the gradient was ramped to 0% A by 5 min and then returned to 95% A. LC-MS/MS peaks were quantified using SCIEX OS 3.1.6.44 software by plotting the sample peak area ratios (analyte/internal) of caffeine and paraxanthine against their respective 11 point calibration standard curves (0.1 ng–1000 ng).

### 2.6 Caffeine metabolic ratio (CMR)

The continuous measure of CMR is the ratio of paraxanthine to caffeine from neonatal plasma samples. CMR was calculated for each participant by dividing the paraxanthine plasma concentration by the corresponding parent drug (caffeine) plasma concentration.

### 2.7 Clinical data collection

Following the informed consent, parents or guardians were asked to complete an optional self-reported ethnicity questionnaire about the patient and biological parents’ ancestral backgrounds. This information was used in the demographic data. Clinical data was longitudinally collected from the SickKids electronic medical record system for participants while inpatient at SickKids. Data collected included demographics, physiology-related variables, drug dosing-related variables, lab-related variables, drug response variables, and concomitant medications.

### 2.8 Statistical analysis

All statistical analyses were conducted using R version 4.4.2. Descriptive statistics summarized participant demographics and sample-level clinical exposures (e.g., clinical status at the time of sample and concomitant medications) within the 24-h window preceding the caffeine dose linked to each plasma sample.

Univariate analyses were conducted to identify clinical covariates that may be associated with CMR. Pearson correlation was used for continuous variables, and Mann-Whitney U or Kruskal-Wallis tests were used for categorical variables. Covariates with suggestive associations (e.g., *p* < 0.05) or plausible biological associations were considered for inclusion in regression models. Pearson correlation was also used to assess the relationship between CMR and both postmenstrual age (PMA) and postnatal age (PNA). Scatterplots of CMR across various ages were generated with overlaid linear regression lines and reported r and p-values. Linear mixed-effects regression models, with participant-level random intercepts to account for repeated measures, were used to assess the association between age and CMR, adjusting for relevant variables. Sensitivity analysis was also performed to determine the robustness of the model. Forest plots were generated to visualize model coefficients and 95% confidence intervals.

A subset of participants had longitudinally collected plasma samples. For these participants, individual-level trajectories of PMA versus CMR were plotted to illustrate intraindividual patterns to better understand interindividual variability.

### 2.9 PBPK modeling

All PBPK simulations were conducted using Simcyp Version 25 (Sheffield, England, United Kingdom). Two published caffeine and preterm models were tested: the default caffeine compound model and default preterm population published by Abduljalil et al. (2020) and the more recently published caffeine and preterm population model by Thomas et al. (2025). Simulations were conducted to match the SickKids patient cohort in terms of median GA, proportion of males and females, dose regimen, dose formulation of caffeine administered, and approximate start and duration of caffeine therapy. The redefine subjects feature was used to simulate maturation of the preterm cohort during caffeine therapy.

The minimum GA in the SickKids cohort was 22 weeks. Unfortunately, neither of the PBPK models tested were developed for preterm patients younger than 25 weeks. Therefore, a second set of simulations were done using a GA of 25 weeks, to assess if simulation at a younger GA would better recover some of the observed data.

### 2.10 Clinical data for PBPK model validation

Observed plasma concentrations from neonates were used for clinical evaluation of the PBPK model predictions. For this purpose, a subset of participants was selected from the original participant cohort. Participants were eligible for inclusion in the model evaluation subset if they received standard caffeine dosing consistent with the simulated regimen (10 mg/kg loading dose followed by maintenance dose of 5 mg/kg daily by intravenous bolus for 100 days PNA). Observed plasma caffeine concentrations from the subset were overlaid on population-level PBPK simulation outputs to enable qualitative comparison with model-predicted median concentrations and 5th–95th prediction intervals.

## 3 Results

### 3.1 Participant characteristics

**FIGURE 1:**
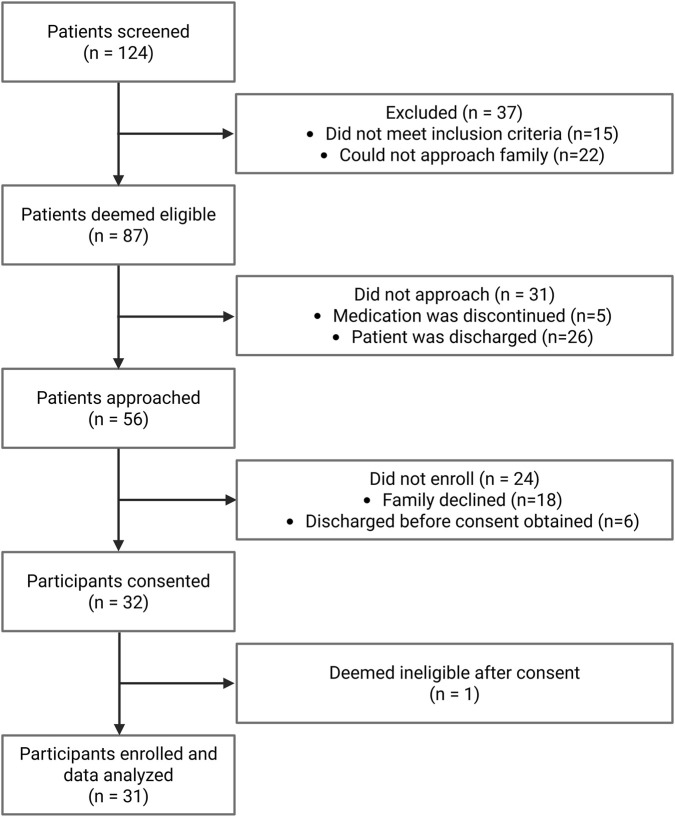
Flow chart of participant recruitment and enrollment.

Between January and June 2025, a total of 124 NICU patients were prescribed caffeine and 87 patients deemed eligible for this study (Figure 1). A total of 31 neonates were then consented and included in the analysis, contributing 186 caffeine pharmacokinetic samples. Participant demographics and baseline clinical characteristics are presented in Table 1. The median GA at birth was 26.3 weeks (range: 22.3–32.9 weeks), and the median birth weight was 926 g (range: 440–2010 g). Participants contributed a median of 7 plasma samples each (range: 1–14 samples), with a median PNA of 27 days (range: 6–75 days). Median PMA at the first sample was 31.1 weeks (range: 25.7–36.0 weeks). A history of gastrointestinal surgery was documented in 35% of participants due to necrotizing enterocolitics (NEC). This is typical of the preterm patient mix at SickKids.

**TABLE 1:** Participant demographics and clinical characteristics.

Characteristic		Count (frequency) or median [range]
Ancestry	African	4 (12.9%)
	Caucasian	8 (25.8%)
	East asian	2 (6.5%)
	Middle eastern	1 (3.2%)
	South asian	6 (19.4%)
	Southeast asian	2 (6.5%)
	2 or more ancestries	6 (19.4%)
	Not reported	2 (6.5%)
Sex	Female	11 (35.5%)
	Male	20 (64.5%)
Gastrointestinal surgery history	Colectomy	1 (3.2%)
	Ileostomy	8 (25.8%)
	Jejunostomy	2 (6.5%)
	No surgery	20 (64.5%)
GA (Weeks)		26.3 [22.3, 32.9]
Birth weight (g)		926 [440, 2010]
Number of plasma samples per participant		7 [1, 14]
PNA at first plasma sample (Weeks)		3.9 [1.0, 10.7]
PMA at first plasma sample (Weeks)		31.1 [25.7, 36.0]

IV, intravenous; GA, gestational age; PNA, postnatal age; PMA, postmenstrual age.

### 3.2 Pharmacokinetic sample characteristics

Sample characteristics and clinical exposures within the 24 h prior to the most recent caffeine dose are summarized in Table 2. The median caffeine dose was 5 mg/kg (range: 3.83–10 mg/kg) and was administered primarily via the intravenous route. Across all pharmacokinetic samples, total parenteral nutrition (TPN) was administered in 79% of samples, while dexmedetomidine and dexamethasone exposure occurred in 14% of samples. Participants had a patent ductus arteriosus (PDA) at the time of sample collection for 29% of samples, and jaundice was present in 81% of samples.

**TABLE 2:** Plasma sample characteristics, clinical exposures, and associated laboratory values.

Variable		Count (frequency) or median [range]
Caffeine route of administration	IV	115 (61.8%)
	Oral	71 (38.2%)
TPN	Yes	147 (79%)
	No	39 (21%)
PDA	Yes	54 (29%)
	No	132 (71%)
Jaundice	Yes	150 (80.6%)
	No	36 (19.4%)
Dexmedetomidine given	Yes	26 (14%)
	No	160 (86%)
Dexamethasone given	Yes	3 (1.6%)
	No	183 (98.4%)
Caffeine dose (mg/kg)		5 [3.83, 10.00]
AST (U/L)		52 [12, 427]
ALT (U/L)		34 [5, 463]
Creatinine (umol/L)		31 [12, 102]
HCT (L/L)		0.33 [0.23, 0.57]
HGB (g/L)		114 [79, 202]
Bilirubin (umol/L)	Direct	20 [1, 217]
	Indirect	19 [1, 150]
	Total	45.5 [5, 339]

TPN, total parenteral nutrition; PDA, patent ductus arteriosus; AST, aspartate aminotransferase; ALT, alanine aminotransferase; HCT, hematocrit; HGB, hemoglobin.

Laboratory parameters collected from 7 days prior to the most recent caffeine dose through the time of sample collection included aspartate aminotransferase (AST), alanine aminotransferase (ALT), creatinine, hematocrit, hemoglobin, and bilirubin fractions. Except for bilirubin values, which were elevated and consistent with the high prevalence of neonatal jaundice, laboratory values generally fell within expected neonatal reference ranges.

### 3.3 Developmental age and caffeine metabolism

**FIGURE 2:**
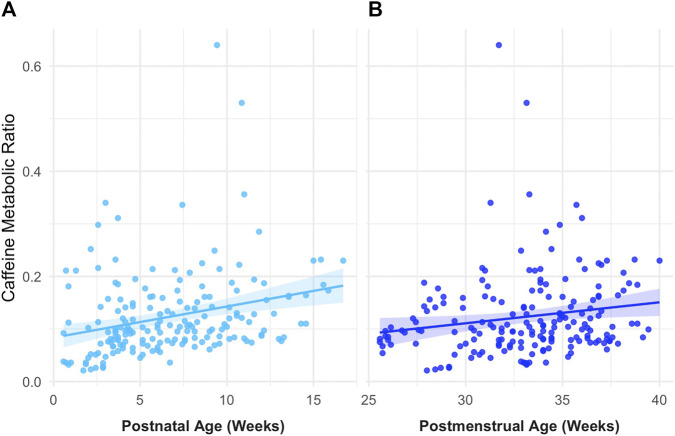
Relationship between participants’ biological variables and caffeine metabolic ratio (CMR). **(A)** Postnatal age (PNA; *r* = 0.27, *p* = 0.0001) and **(B)** postmenstrual age (PMA; *r* = 0.17, *p* < 0.05). Each point represents a single plasma sample. Solid lines represent linear regression fits with shaded 95% confidence intervals.

**FIGURE 3:**
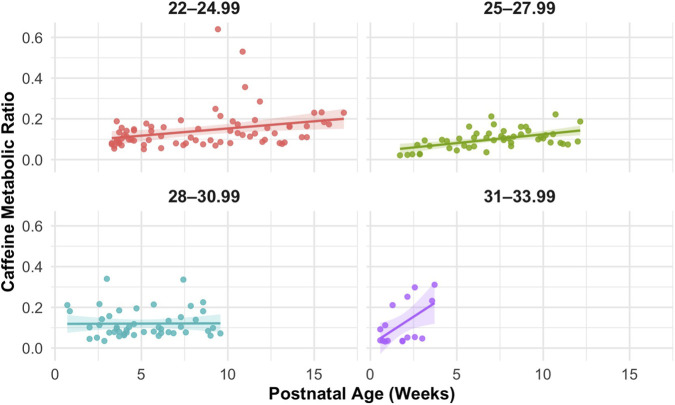
Postnatal age (PNA) vs. caffeine metabolic ratio (CMR) stratified by gestational age (GA) at birth. Scatterplots show CMR for individual plasma samples grouped into GA categories: 22–24.99 weeks (*r* = 0.3, *p* = 0.01), 25–27.99 weeks (*r* = 0.5, *p* = 0.0001), 28–30.99 weeks (*r* = 0.01, *p* = 0.95), and 31–33.99 weeks (*r* = 0.55, *p* < 0.05). Solid lines represent linear regression fits with shaded 95% confidence intervals. Differences in slopes highlight that the combination of GA and PNA (i.e., PMA) better reflects developmental changes in CYP1A2 activity than PNA alone.

In univariate analyses, both PNA and PMA demonstrated statistically significant positive correlations with CMR. As shown in Figures 2A,B, CMR increased with both PNA (*r* = 0.27, *p* < 0.001) and PMA (*r* = 0.20, *p* = 0.02), although the association with PNA was stronger. When the relationship between PNA and CMR was stratified by GA at birth (Figure 3), distinct PMA-CMR trajectories were observed across GA categories (22–24.99, 25–27.99, and 31–33.99 weeks). Although CMR tended to increase with PNA in all groups, the slope of this increase differed by GA category.

**FIGURE 4:**
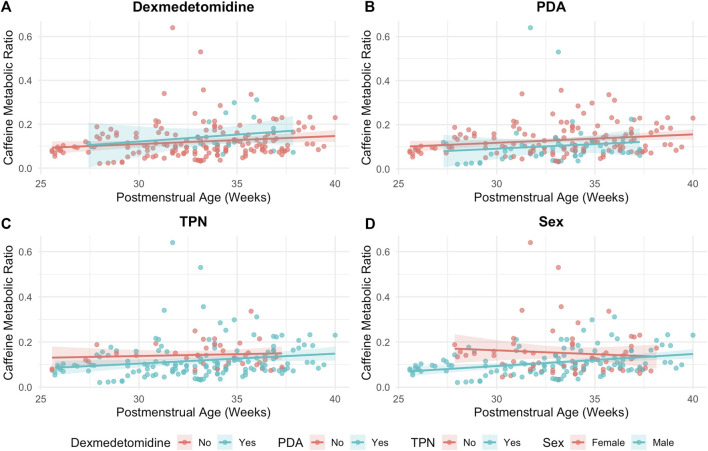
Postmenstrual age (PMA) vs. caffeine metabolic ratio (CMR) stratified by clinical exposures. **(A)** Dexmedetomidine exposure within 24 h prior to the most recent caffeine dose, **(B)** presence of a patent ductus arteriosus (PDA), **(C)** total parenteral nutrition (TPN) administration within 24 h prior to the most recent caffeine dose, and **(D)** sex. Each point represents a single plasma sample. Solid lines represent linear regression fits with shaded 95% confidence intervals.

Several clinical exposures were found to significantly modify the relationship between PMA and CMR (Figure 4). Infants with a PDA had significantly lower CMR values compared with those without a PDA (*p* < 0.001) (Figure 4B). Similarly, infants receiving TPN demonstrated lower CMRs (*p* < 0.001) (Figure 4C). CMR differed by sex, with males and females showing distinct trajectories (*p* < 0.01) (Figure 4D). In contrast, recent exposure to dexmedetomidine was not significantly associated with CMR (*p* = 0.09) (Figure 4A).

### 3.4 Mixed-effects regression model and sensitivity analyses

**FIGURE 5:**
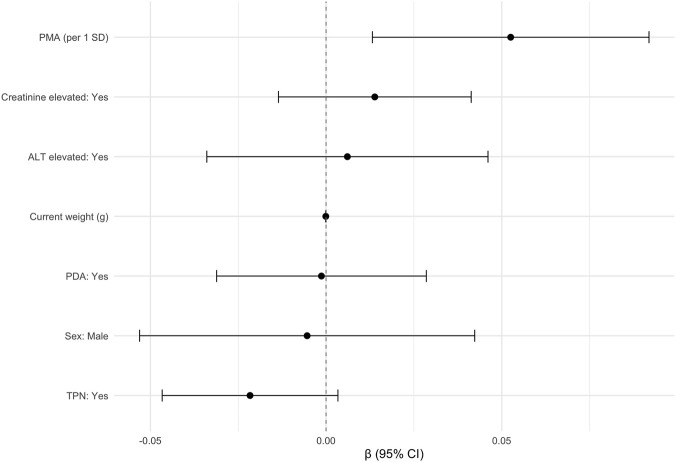
Forest plot of fixed effects from the primary mixed-effects regression model. This model included PMA (per 1 SD) and clinically relevant covariates (current weight, sex, PDA, TPN, elevated ALT, and elevated creatinine). Each point represents the estimated regression coefficient (*β*), with horizontal lines indicating 95% confidence intervals.

To further investigate the effect of age on CYP1A2 enzyme ontogeny via CMR, the relationship between PMA and CMR was modeled using a linear mixed-effects regression model (Figure 5). PMA was selected as the primary age variable as it integrates both gestational maturity at birth and postnatal development. PMA was scaled (per 1 SD increase) to facilitate interpretability and comparability of effect sizes across predictors.

Demographic characteristics, clinical characteristics, and exposure to concomitant medications were screened for univariate associations with CMR (Supplementary Table S1). Variables demonstrating statistically significant associations or biological plausibility were included as covariates in the primary mixed-effects model. Random intercepts were included to account for repeated measures within individuals.

In the primary regression model, increasing PMA (per 1 SD) was significantly associated with higher CMR (*β* = 0.053, *p* = 0.01; Figure 5), consistent with univariate analyses (Figure 2B). This corresponded to an estimated increase of approximately 0.05 in CMR for every 3.4-week increase in PMA when all other covariates were held constant. Weight at the time of sampling demonstrated a small negative association with CMR (*β* = −0.000066, *p* = 0.016). However, weight and PMA are strongly collinear (*r* = 0.89), indicating substantial overlap between these variables.

Sex, TPN exposure, PDA status, and elevations in ALT or creatinine were not significantly associated with CMR in the adjusted model. Sensitivity analyses demonstrated that the association between PMA and higher CMR was directionally consistent across all regression models, although statistical significance was lost in several subset analyses (Supplementary Figure S1). When weight was excluded from the model, the association between PMA and CMR remained positive.

### 3.5 Longitudinal analyses

**FIGURE 6:**
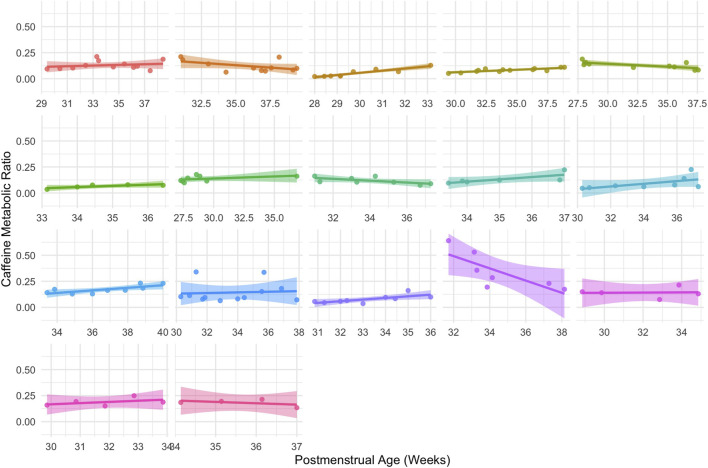
Longitudinal within-subject trajectories of postmenstrual age (PMA) vs. caffeine metabolic ratio (CMR). Each panel represents an individual participant with multiple plasma samples collected over time. Solid lines indicate linear regression fits with shaded 95% confidence intervals. Most participants show an upward trajectory of CMR with increasing PMA.

After establishing PMA as the primary predictor of interindividual variability in CMR, within-subject longitudinal trajectories were examined among neonates with repeated plasma samples. Longitudinal plots demonstrated that CMR generally increased with increasing PMA within the same neonate, as observed in 8 of 14 neonates (Figure 6). However, despite increasing PMA, four neonates displayed a downward trajectory in CMR, and one neonate showed minimal change in CMR across repeated samples. No consistent clinical pattern was observed among these five neonates.

### 3.6 PBPK model fit and validation

**FIGURE 7:**
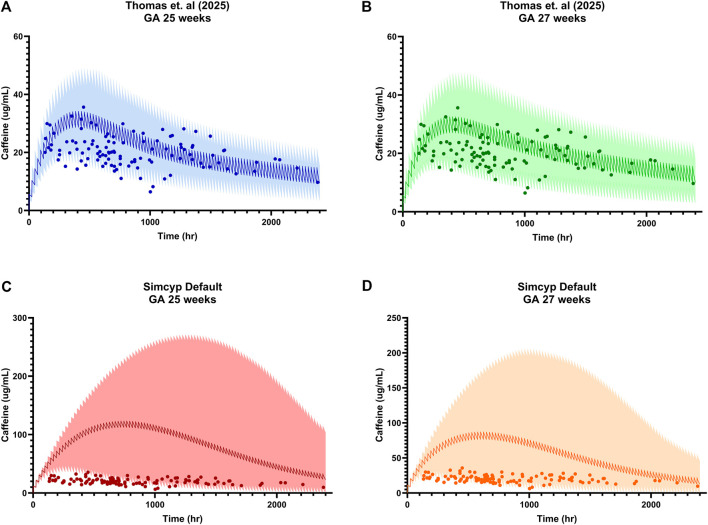
Comparison of observed neonatal caffeine concentrations with physiologically based pharmacokinetic (PBPK) model simulations at 25 and 27 weeks gestational age (GA). **(A)** Thomas et al. (2025) ontogeny model, GA 25 weeks (blue); **(B)** Thomas et al. (2025) ontogeny model, GA 27 weeks (green); **(C)** Abduljalil et al. (2020) GA 25 weeks (red); and **(D)** Abduljalil et al. (2020) GA 27 weeks (yellow). Observed plasma caffeine concentrations from our clinical cohort are shown as points, with simulated mean concentration–time profiles depicted as solid lines and 5th–95th percentile prediction intervals shown as shaded regions. Time represents time of plasma sample collection relative to birth. Simulations reflect standard clinical dosing: 5 mg/kg maintenance dosing intravenously every 24 h starting at birth.

To evaluate the performance of existing neonatal PBPK models, observed plasma concentrations from our clinical cohort were overlaid onto simulated caffeine concentration-time profiles for 25- and 27-weeks GA (Figure 7). Using the Abduljalil et al. (2020), simulations markedly overpredicted systemic caffeine exposure at both GAs (Figures 7C,D). Predicted peak concentrations and overall exposure were substantially higher than those observed clinically, and the 5th–95th percentile prediction intervals were wide and shifted upward relative to the data. Most observed concentrations clustered well below the simulated median and frequently near or beneath the lower prediction bound, suggesting overestimation of exposure and underestimation of caffeine clearance in the default neonatal model.

In contrast, simulations generated using the Thomas et al. (2025) model, which incorporates an updated CYP1A2 ontogeny function, updated glomerular filtration rate (GFR) function, updated age-weight function, and updated caffeine model demonstrated improved concordance with observed data (Figures 7A,B). Predicted mean concentration-time profiles more closely aligned with the central tendency of the clinical cohort, and a greater proportion of observations fell within the 5th–95th percentile prediction intervals. However, even with the updated model, observed concentrations tended to occupy the lower half of the simulated variability range.

## 4 Discussion

In this study, we provide the first *in vivo* evidence that CYP1A2 is expressed and quantifiable in even the most preterm neonates, as demonstrated by detectable paraxanthine formation and age-associated increases in CMR. A positive correlation was observed between age and CMR, indicating that caffeine metabolism increases with developmental age which is consistent with CYP1A2 maturation (Figure 2). Although CYP1A2 activity was low overall, measurable inter- and intra-individual variability in CMR indicated that hepatic caffeine metabolism is not uniform across neonates. Notably, our cohort was more diverse in ancestry (Table 1) compared to prior neonatal pharmacokinetic studies (Dai et al., 2024; Gao et al., 2021; He et al., 2021; Ide et al., 2024; Taguchi et al., 2021), which may further contribute to observed variability and improve generalizability of these findings. Generally, more premature neonates demonstrated a delay in CYP1A2 ontogeny compared to neonates of the same PNA (Figure 3). In longitudinal within-subject analyses, 8 of 14 infants showed rising CMR with age, while a few exhibited flat or downward trajectories (Figure 6), indicating an overall maturational increase in CYP1A2 activity over time but with notable variability between individuals (Figure 5). Consistent with these findings, PBPK simulations suggested that existing neonatal ontogeny functions may underestimate caffeine clearance in extremely preterm infants (Figure 7), further supporting earlier or more variable emergence of CYP1A2 activity than currently modeled. Together, these findings reveal age-related trends in CYP1A2 activity with substantial interindividual variability, suggesting that enzyme ontogeny is a dynamically regulated and clinically sensitive process.

Historically, neonatal caffeine clearance was viewed as renal excretion-dominated with unchanged drug and negligible hepatic metabolism due to observations of prolonged half-life, high urinary recovery of parent drug, and frequent absence of measurable paraxanthine in early life (Aldridge et al., 1979; He et al., 2021; Kennedy et al., 2004; Lee et al., 1997). *In vitro* studies have reinforced this as demonstrated by delayed hepatic expression of CYP1A2 relative to other enzymes (Sonnier and Cresteil, 1998) and low CYP1A2 abundance (Subash and Prasad, 2024). Our study detected *in vivo* paraxanthine, with measurable CMR which adds nuance to this perspective. We demonstrated that CYP1A2 activity appears low but is present during early postnatal development. The observation of measurable caffeine and paraxanthine levels in extremely preterm infants suggests that CYP1A2 regulation in early life may be more dynamic than previously thought (Hines and McCarver, 2002; van den Anker et al., 2018; van Groen et al., 2021). Although fetal CYP1A2 expression is traditionally considered absent, this may reflect transcriptional repression in the hypoxic intrauterine environment (Denison and Nagy, 2003; Nebert et al., 2004; Vorrink and Domann, 2014; Zhang et al., 2022). Following preterm birth, abrupt exposure to oxygen and xenobiotics may partially relieve this repression and permit earlier CYP1A2 expression and activity, providing a biologically plausible explanation for detectable CMR values in preterm infants despite expectations of minimal or absent activity (Denison and Nagy, 2003; Hines and McCarver, 2002). These findings further indicate that CYP1A2 ontogeny is not uniform across neonates and may be highly sensitive to clinical exposures in the NICU. Pharmacogenetic variation may represent an additional source of unexplained variability (He et al., 2021; Sachse et al., 1999; Thorn et al., 2012). Although genotype-phenotype relationships were not evaluated in the present analysis, we have collected genetic data from this cohort and will expand on this in future studies.

Across univariate and multivariable analyses, PMA emerged as the most informative developmental predictor of CMR (Figure 5). Although both PNA and PMA were positively associated with CMR, stratified analyses demonstrated that neonates with similar PNA but differing gestational maturity exhibited distinct CMR trajectories. This indicates that PMA better captures maturational changes than PNA alone and supports the concept that enzyme ontogeny reflects the combined contributions of prenatal and postnatal development rather than time since birth alone (Ide et al., 2024). PMA integrates both components and therefore more accurately captures the biological maturation of hepatic metabolic capacity. These findings are consistent with prior pharmacokinetic studies showing that GA at birth influences drug metabolism early in life and that postnatal maturation accelerates metabolic capacity over time. This also further extends the utility of CMR as a functional biomarker of CYP1A2 activity through plasma pharmacokinetic samples rather than urinary clearance alone.

Despite PMA being the dominant predictor of CMR in the mixed-effects model, the overall effect size was small and residual variability in CMR remained unexplained. This is an important finding, as it highlights that developmental age alone is insufficient to fully account for interindividual differences in neonatal caffeine metabolism and suggests that additional clinical or genetic factors may modulate CYP1A2 activity in early life. In our data, age explained only a portion of the variance. Univariate analyses suggested lower CMR with TPN and PDA and sex-related differences (Supplementary Table S1). In the adjusted mixed-effects model, PMA remained the primary predictor and these clinical covariates were no longer significant, likely reflecting collinearity, sample size, and overlapping biology. The negative coefficient for weight in the adjusted model, despite strong collinearity with PMA (*r* = 0.89), further underscores that developmental age rather than weight better captures the relevant developmental signal for CYP1A2 activity in early life, consistent with existing literature (Allegaert and van den Anker, 2014; Anderson and Holford, 2009; van den Anker et al., 2018). These trends demonstrate the complexity of disaggregating the effects of ontogeny from environment and illness on drug metabolism in a critically ill population. For instance, one infant was documented to have Alagille syndrome which impairs hepatic function, illustrating that hepatic function may alter CYP1A2 activity (Kamath et al., 2010; Verbeeck, 2008). Some infants also had comorbidities including PDA, which can alter hepatic blood flow and thus, may affect drug-metabolizing enzyme activity (Mifflin et al., 2025; Padavia et al., 2024).

Moreover, inter- and intraindividual variability in CMR observed indicates that CYP1A2 ontogeny in neonates is very heterogenous and influenced by factors beyond age alone. The substantial interindividual variability observed further indicates that current PBPK models do not fully capture neonatal physiology and drug disposition, particularly in extremely preterm infants. Together, these results underscore the limitations of applying generalized preterm infant PBPK models derived from *in vitro* ontogeny data (Abduljalil et al., 2020; Salem et al., 2014) and support the development of bespoke, population-specific models that incorporate clinically relevant modifiers (e.g., critical illness and ontogenies based on GA specific trajectories). The reasoning for this is we can model concomitant medications with PBPK models, so you can account for that without changing the population model. However, the impact of critical illness and maturation based on GA at birth would be population changes that are more specific to the population of interest. By characterizing both the average maturational trajectory and the variability in CYP1A2 activity, our findings provide clinically derived data that may help identify which developmental and clinical factors warrant further evaluation and integration into PBPK models. Additionally, incorporating an *in vivo* derived CYP1A2 ontogeny function derived from preterm infants into PBPK models may improve the accuracy of CYP1A2 substrate predictions in this population, similar to what has been shown for pediatric PBPK models (Codaccioni et al., 2024; Upreti and Wahlstrom, 2016). Additionally, for the purposes of using PBPK models to develop dosing tools for NICU preterm patients, it is especially important to develop a PBPK model that reflects the patient population. This is of particular importance in extremely preterm or clinically complex infants, where traditional weight-based approaches underperform (Leong et al., 2012).

Model-based simulations provided a practical test of whether existing neonatal PBPK models capture the average change and variability in CYP1A2 activity (Figure 7). It is important to consider that the Abduljalil et al. (2020) was developed to represent a healthy preterm population using generalized maturation functions, whereas the Thomas et al. (2025) model was parameterized using data from very low birth weight and clinically complex infants (e.g., impaired renal function). The improved concordance of the Thomas model in our cohort may therefore reflect better alignment with the physiology of critically ill neonates, who comprise the majority of preterm infants in NICUs receiving caffeine. Comparison of observed intravenously dosed caffeine concentrations from neonatal plasma with simulated PBPK profiles demonstrated systematic overprediction of exposure by default Simcyp neonatal ontogeny functions and underestimation of clearance in current models. Although neonates with gastrointestinal surgery were included in our clinical cohort, altered drug absorption due to surgery is unlikely to have contributed to low systemic caffeine concentrations observed, as caffeine is primarily absorbed in the proximal small intestine (Aranda and Beharry, 2020; Olivares-Morales et al., 2015; Perera et al., 2012). Further, PBPK comparisons were restricted to intravenous dosing, differences between observed and predicted concentrations are unlikely to reflect variability in absorption. Instead, these discrepancies likely relate to model assumptions on hepatic clearance, growth trajectories for preterm patients, and GFR maturation trajectories.

Alternative ontogeny parameterizations by Thomas et al. (2025) improved concordance between predicted and observed concentrations but some variability remained unexplained. Values frequently occupied the lower range of the predicted variability, suggesting residual overprediction of exposure in a subset of infants. One reason may be our clinical cohort which included extremely preterm infants (<25 weeks GA), extending beyond the GA represented in either model and potentially reflecting CYP1A2 ontogeny patterns not yet adequately characterized. In addition, polypharmacy is common in NICU settings, with infants frequently receiving multiple concomitant medications (Krzyżaniak et al., 2016), including diuretics that may influence renal function. Extremely preterm infants (22–24 weeks GA) also have markedly reduced renal clearance, minimal adipose tissue, altered total body water composition, and immature protein binding, which could all alter caffeine disposition and contribute to divergence between observed and predicted concentrations.

Furthermore, the Thomas model employed a minimal distribution model which may not fully capture dynamic changes in volume of distribution, protein binding, hepatic blood flow, and enzyme activity in this population. These factors, together with the unique body composition of preterm infants who may only have 1.4%–1.7% body fat (Salas et al., 2021), can limit the ability of existing parameterizations to accurately reflect drug disposition in the most immature neonates.

Interestingly, the default Simcyp neonatal model predicted maximum systemic caffeine concentrations between 200–270 μg/mL, whereas both our observed data and previously published neonatal pharmacokinetic studies consistently report maximum concentration values in the range of 40–50 μg/mL (Dai et al., 2024; Gao et al., 2021; Ide et al., 2024; Taguchi et al., 2021). The Thomas et al. (2025) model demonstrated closer alignment with observed central tendency, with predicted maximum caffeine concentration values approximately 30–40 μg/mL, however, the upper 95th percentile extended to 40–50 μg/mL. These findings are particularly striking given that both the default Simcyp model and the upper percentile of the Thomas model predict systemic caffeine concentrations beyond the therapeutic range of 5–30 μg/mL, and the default Simcyp model exceedingly surpasses the toxic threshold of 50 μg/mL (Sugino et al., 2023).

This is critically important if PBPK models are being used to inform dosing decisions, as inaccurate exposure predictions could lead to inappropriate dose selection. Achieving adequate systemic exposure is essential not only for the short-term management of apnea of prematurity, but also for optimizing long-term neurodevelopmental outcomes (Ostrem et al., 2025). Thus, these results suggest that existing neonatal PBPK models may inadequately represent CYP1A2-mediated drug metabolism and subsequent caffeine clearance in extremely preterm infants. Together, these findings support the development of bespoke PBPK frameworks tailored to extremely low GA or very low birth weight infants, rather than reliance on generalized preterm models extrapolated downward from older populations. Integration of clinically derived *in vivo* ontogeny data such as those generated in this study may improve predictive performance neonatal PBPK frameworks for future precision dosing.

The present study does also have some limitations that should be considered when interpreting these findings. First, the relatively small sample size reflects the inherent challenges of conducting pharmacokinetic research in critically ill neonates, where ethical considerations, clinical instability, limited blood volume, and reliance on scavenged sampling constrain recruitment and sampling frequency (Allegaert and van den Anker, 2014; Kearns et al., 2003). These factors limit statistical power, reduce the feasibility of uniform longitudinal sampling, and may restrict the ability to fully disaggregate CYP1A2 ontogeny from interindividual variability.

Another limitation is that CMR is an indirect marker of CYP1A2 activity and may be influenced by factors beyond hepatic metabolism. In neonates, developmental changes in renal function, and fluid balance may alter caffeine and metabolite concentrations independently of hepatic enzyme activity. Variation in availability of metal co-factors such as iron may influence CYP enzyme function, introducing additional sources of variability into CMR-based estimates of CYP1A2 activity (Shteyer et al., 2015). In addition, there is no published data clarifying whether paraxanthine clearance in neonates is formation rate-limited or elimination rate-limited. Without understanding the dominant kinetic process governing paraxanthine disposition in this population, interpretation of CMR as a direct surrogate of CYP1A2 activity remains constrained.

An additional limitation is that inflammatory markers were not assessed in this study. Inflammation has been associated with downregulation of CYP1A2 in adults, likely through cytokine-mediated suppression of hepatic enzyme expression (Frye et al., 2002; Lenoir et al., 2021). Inflammatory signaling may also contribute to the observed variability in neonatal CYP1A2 activity since infants in the NICU may experience inflammation following infection or surgery. However, relevant inflammatory markers such as recent C-reactive protein (CRP) measurements were not available for more than half of the neonates in this study. Consequently, inflammation could not be incorporated as a covariate in mixed-effects regression models. Future studies should incorporate prospective and longitudinal assessment of inflammatory status to better characterize its role in modulating CYP1A2 activity in preterm neonates. This may include CRP measurements along with specific biomarkers of inflammatory signaling and hepatic metabolism.

Further, existing preterm ontogeny functions implemented in commonly used PBPK platforms do not extend below a GA of 25 weeks (Abduljalil et al., 2020; Salem et al., 2014; Thomas et al., 2025). As a result, extremely preterm neonates may exhibit lower renal clearance and distinct distributions which are not captured by models parameterized in older neonates. Minimal or reduced structural PBPK frameworks may not fully capture these distributional nuances. Many neonatal models also rely on stepwise extrapolation from adult or older pediatric physiology. This limitation underscores the importance of have an enriched empirical *in vivo* data set from extremely preterm neonates to inform and recalibrate neonatal PBPK models.

Finally, the absence of pharmacogenomic data in this study limits mechanistic inference regarding genetic contributors to interindividual variability in caffeine metabolism. Genetic data have been collected but remain pending analysis and therefore could not be incorporated alongside ontogeny assessments in this work. As a result, the extent to which genetic variation modulates developmental trajectories of CYP1A2 activity could not be evaluated, but observed variability in CMR may reflect a combination of maturational, clinical, and genetic influences that warrant further investigation.

One important next step is to determine whether the observed CYP1A2 activity is sufficient to influence caffeine clearance and dosing in the clinical setting. Although overall activity was low, the presence of measurable variability indicates that hepatic metabolism may contribute to interindividual differences in drug response. Our future analyses will examine the relationship between CYP1A2 activity (using CMR as a proxy) and clinically relevant outcomes such as apnea, acid reflux, and tachycardia. These analyses will determine whether observed enzyme activity leads to meaningful differences in drug exposure and clearance that may warrant dosing adjustments in this population.

Overall, this study demonstrates that CYP1A2 activity is detectable, developmentally regulated, and clinically modulated in extremely preterm neonates. CYP1A2 ontogeny follows a maturational trajectory best captured by PMA, yet exhibits substantial interindividual variability influenced by clinical exposures and potentially genetic factors. These findings challenge simplified weight-based dosing paradigms and provide mechanistic insight into the heterogeneity of caffeine disposition in critically ill infants. Our study further highlights the importance of generating empirical clinical pharmacokinetic data directly from the neonatal population to inform mechanistic modeling. Reliance on extrapolated ontogeny functions or generalized preterm assumptions may inadequately represent the physiology of critically ill preterm infants. By integrating clinically derived *in vivo* data into PBPK frameworks, bespoke models of neonatal drug disposition can be developed to better reflect the average hospitalized infant receiving multiple concomitant medications.

Improved mechanistic PBPK models carry meaningful translational impact. Accurate characterization of hepatic and renal clearance in infants may enable dosing strategies grounded in developmental physiology rather than empirical weight-based scaling alone. By integrating robust clinical pharmacokinetic data with refined PBPK models, this combined framework can be translated into more individualized clinical dosing guidelines tailored to developmental age, organ maturation, and illness severity. Such model-informed recommendations could be operationalized by neonatologists and hospital pharmacies to guide dosing decisions at the bedside, a paradigm shift to physiologically informed, precision-based therapeutic strategies in the NICU. Ultimately, rigorous study of drug disposition and hepatic enzyme ontogeny in extremely preterm infants advances pharmacoequity for a historically understudied population and lays critical groundwork for future precision therapeutics in neonatal care.

## Data Availability

The raw data supporting the conclusions of this article will be made available by the authors, without undue reservation.
