# Supplementary material for: Developmental age and clinical illness drive in vivo variability of CYP1A2 ontogeny and caffeine metabolism in preterm neonates
Source: Front Pharmacol. 2026 May 20;17:1823969. doi: 10.3389/fphar.2026.1823969 (PMC13229812; doi:10.3389/fphar.2026.1823969)
Supplement: Supplementary file 1 [file DataSheet1.docx]

Supplementary Material

**Supplementary Table 1.** Univariate associations with CMR

| **Variable** | **Statistical Test** | **Statistic** | **df** | **p-value [95% CI]** | **Significance** |
| --- | --- | --- | --- | --- | --- |
| PMA | Pearson | t = 2.339 | 184 | 0.02041  [0.0267, 0.3063] | Y |
| PNA |  | t = 3.8574 |  | 0.0001584  [0.1350, 0.4016] | Y |
| Caffeine Dose |  | t = 0.42051 |  | 0.6746  [-0.1741, 0.1134] | NS |
| Weight |  | t = 2.0093 |  | 0.04596  [0.0027, 0.2844] | Y |
| Length |  | t = 1.7645 |  | 0.0793  [-0.0151, 0.2679] | NS |
| Sex | Mann-U Whitney | W = 4791 | N/A | 0.008386 | Y |
| Route of Administration |  | W = 3508 |  | 0.1076 | NS |
| Dexmedetomidine |  | W = 1210 |  | 0.09083 | NS |
| Dexamethasone |  | W = 355 |  | 0.3871 | NS |
| TPN |  | W = 3837.5 |  | 0.0005351 | Y |
| Breast milk |  | W = 3871.5 |  | 0.8022 | NS |
| Jaundice |  | W = 2643.5 |  | 0.3794 | NS |
| PDA |  | W = 4778.5 |  | 0.00002302 | Y |
| Elevated AST |  | W = 2911.5 |  | 0.2665 | NS |
| Elevated ALT |  | W = 1750.5 |  | 0.0485 | Y |
| Elevated HCT |  | W = 2636 |  | 0.2738 | NS |
| Elevated HGB |  | W = 3055.5 |  | 0.9523 | NS |
| Elevated Creatinine |  | W = 4557 |  | 0.04968 | Y |


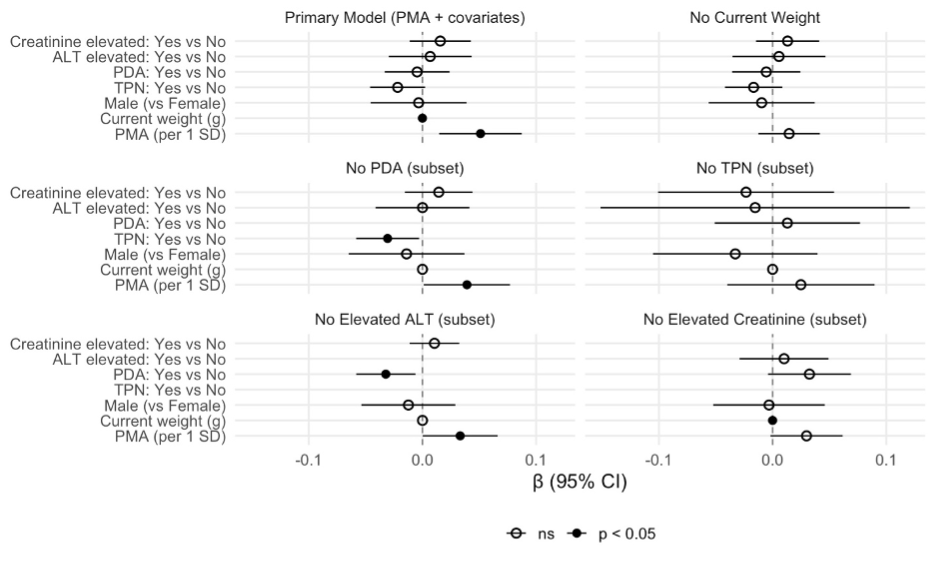


**Supplementary Figure 1.** Forest plot of fixed effects with 95% confidence intervals across the primary and sensitivity mixed-effects regression models. Filled circles indicate *p*<0.05; open circles indicate non-significance.
